# Sexuality Education for Youth and Adolescents in the Middle East and North Africa Region: A Window of Opportunity

**DOI:** 10.9745/GHSP-D-23-00282

**Published:** 2024-02-28

**Authors:** Doaa Oraby

**Affiliations:** a Independent public health consultant, Cairo, Egypt.

## Abstract

The enabling role of Islam in the notion of sexuality education can serve as a window of opportunity for implementing culturally and contextually relevant sexuality education programs in the Middle East and North Africa region.

## Key Messages

- Adolescents and youth require sexual and reproductive health information, education, and services globally to see positive health outcomes. This is also true of Muslim youth in the Middle East and North Africa (MENA) region.
- The controversial nature of sexuality education in the MENA region is not a result of Islamic doctrine but rather the result of cultural sexual taboos being misinterpreted as grounded in Islam.
- The Western approach to sexuality education should be tailored and culturally adapted to the MENA region because of the differences in history, religion, and culture.
- Incorporating sexuality education within religious and cultural frameworks is not an easy task and is best achieved through an ongoing process that is open to change and flexible to adaptation based on evidence-based findings.

## SEXUALITY EDUCATION FOR YOUTH AND ADOLESCENTS IN THE MIDDLE EAST AND NORTH AFRICA REGION

The Middle East and North Africa (MENA) is home to 80 million youth, and its young adult population makes up 10% of the world’s population. Concurrently, the region is home to more than half of the world’s refugees, with more than one-third of young people living in fragile and conflict-affected countries.^1^ These conflict situations, with prevalent poverty and limited economic opportunities in the region, heighten the vulnerability of young people to HIV, sexually transmitted infections (STIs), unintended pregnancies, and unsafe abortion. In the Arab region, 2 in 5 pregnancies are still unintended and one-half of unintended pregnancies end in abortion.^2^ This vulnerability is further aggravated by the prevalent violence and collapsed health systems in the affected countries.^3^ The situation is even worse for adolescent girls who face complex challenges that alter the course of their development for their entire lives.^4^

Despite having the lowest HIV prevalence in the world (less than 0.1%), the MENA region is one of few regions where the HIV epidemic has not waned and is increasing with a 31.0% increase of HIV infections since 2001.^5^ Youth in the MENA region are at particular risk of HIV due to challenges in accessing services, inhibition about discussing both sexual and reproductive health (SRH), and lack of credible sex education sources.^6^ These demographic and socioeconomic transformations, coupled with evidence about the rise of HIV and STIs among young people in the region, led to concerns about protecting these vulnerable young people against these threats. The Sustainable Development Goals target 3.7 calls on countries to ensure universal access to SRH services, including information and education, by 2030.^7^ This viewpoint focuses on how young people in the MENA region obtain their SRH information, barriers they experience in accessing this information, and how their access can be increased to improve their overall health and well-being.

In the MENA region, young people become sexually active without being provided accurate information on their SRH. Parents and other adults feel embarrassed to discuss sexuality with their children and fear that discussion on sexuality may cause sexual arousal and subsequent sexual practice. They usually surrender their responsibility to schoolteachers, who feel embarrassed and feel that this responsibility lies with the family and not the school. Accordingly, young people resort to the Internet, online pornography, and peers as sources of SRH information,^8^ which are often inaccurate and potentially harmful to equitable gender norms.

The culture of silence surrounding sexual health in the MENA region increases the vulnerability of young people to HIV, STIs, unintended pregnancies, and unsafe abortion.^6^^,^^9^ The situation is further aggravated by the current political turmoil and conflict zones where young people may resort to transactional sex for survival. Thus, sexuality education of young people in the MENA region is timely and critical yet not easy to achieve.

The MENA region is not homogenous, and countries vary dramatically in wealth, educational attainment, social norms, cultural values, and social and economic policies, including those that influence sexuality education or the lack thereof. Although a few countries have some form of sexuality education, it is inadequate overall. Countries in the MENA region, with common historical, linguistic, and religious characteristics, experience similar bottlenecks regarding the implementation of sexuality education programs. In these countries, sexuality education is perceived as a manifestation of the pervasive influence of the Western social norms that clash with Muslim values and norms about sex, especially the topics of premarital sex and homosexuality, which are frowned upon by conservative communities.^10^

## A WINDOW OF OPPORTUNITY

Most of the opponents of sexuality education build their arguments on the fact that Islam, the prevalent religion in the region, views sex before marriage as sinful and expects young people to remain nonsexual after puberty until marriage. Thus, they believe there is no room to accommodate sexuality education conceptualized in the Western context. Studies have highlighted that the controversial nature of sexuality education for young people is not the result of Islamic doctrine but rather of perceiving sex as a cultural taboo. Accordingly, barriers related to sexuality education are not grounded in Islam but in the culture of silence.^11^^,^^12^ Islam recognizes the power of sexual need and encourages age-specific education about matters related to sex according to Islamic values and principles.^7^ Quran, the holy book of Islam, emphasizes the importance of acquiring knowledge that is not limited to religious knowledge: “Are those who know equal to those who do not know?” (The Quran, 39:9). Hadith, the sayings of the Prophet Muhammad, also praised the seeker of knowledge. Furthermore, in the days of the Prophet Muhammad, Muslim men and women were never too shy to ask him questions related to sexual life.^13^

Islam acknowledges human nature—that what is promoted religiously is not necessarily what is practiced and that risky behaviors that are not allowed by Islam are indeed practiced.^14^ Furthermore, the 2 key tenets of Islam that support harm reduction programs in Islamic countries—doing no harm to oneself or others and the worst harm is eliminated by a lesser harm—accommodate sexual education programs aimed at youth to reduce negative health consequences. In 2005, the Islamic Republic of Iran introduced HIV harm reduction interventions to reduce the harm related to injecting drug use and to prevent the spread of HIV among injecting drug users. The interventions included outreach activities for injecting drug users and providing them with sterile syringes. Illicit drug use is prohibited in Islam. Yet, when viewed through the Islamic tenets, harm reduction programs are permissible and provide a practical solution to a problem that could result in far greater damage to society if left unaddressed.^15^^,^^16^

The enabling role of Islam in the notion of sexuality education can serve as a window of opportunity for the implementation of relevant programs in the MENA region and other Islamic countries. Efforts should be made to develop tailored programs adapted to the religious values and local context while incorporating the Western expertise and relevant lessons learned and best practices. Given that “no one size fits all,” sexuality education programs should be adapted to the local context of the different countries of the MENA region.

In Islam, sexuality education seeks to guide individuals to channel their sexual needs and desires responsibly and in an honorable manner. Hence, the focus should be on the Islamic virtues of decency, modesty, chastity, and sexual responsibility, in addition to highlighting the concept of family life and SRH needs. Given the powerful impact of religious leaders who play a critical role in Muslim culture, hosting a founding conference on sexuality education with theological institutions should be considered a preparatory step.

## Notes

**Peer Reviewed**

**First published online:** January 31, 2024.

## References

[1] UNICEF. *MENA Generation 2030: Investing in Children and Youth Today to Secure a Prosperous Region Tomorrow.* UNICEF Regional Office for the Middle East and North Africa; 2019. Accessed January 10, 2024. https://www.unicef.org/mena/reports/mena-generation-2030

[2] United Nations Population Fund Arab States Regional Office (UNFPA/ASRO). *Addressing Unintended Pregnancy in the Arab Region*. Middle East North Africa Health Policy Forum/UNFPA/ASRO; 2018. Accessed January 26, 2024. https://arabstates.unfpa.org/sites/default/files/pub-pdf/addressing_unintended_pregnancy_in_the_as_report.pdf

[3] International Labor Office (ILO). *Promoting a Rights-Based Approach to Migration, Health and HIV and AIDS: A Framework for Action.* ILO; 2017. Accessed January 10, 2024. https://www.ilo.org/wcmsp5/groups/public/---dgreports/---gender/documents/publication/wcms_605763.pdf

[4] United Nations Population Fund (UNFPA). *In Her Words: Stories of Adolescent Girls Living in Humanitarian Settings in the Arab Region.* UNFPA Regional Humanitarian Hub for Syria and the Arab States; 2021. Accessed January 10, 2024. https://palestine.unfpa.org/sites/default/files/pub-pdf/in_her_words_-_english_-_final.pdf

[5] Joint United Nations Programme on HIV/AIDS (UNAIDS). *The Gap Report.* UNAIDS; 2014. Accessed January 10, 2024. https://www.unaids.org/sites/default/files/media_asset/UNAIDS_Gap_report_en.pdf 12349391

[6] Kteily-Hawa R, Hawa AC, Gogolishvili D, et al. Understanding the epidemiological HIV risk factors and underlying risk context for youth residing in or originating from the Middle East and North Africa (MENA) region: a scoping review of the literature. PLoS One. 2022;17(1):e0260935. 10.1371/journal.pone.0260935. 34995320 PMC8741013

[7] United Nations (UN). Department of Economic and Social Affairs. Population Division. *Family Planning and the 2030 Agenda for Sustainable Development: Data Booklet.* UN; 2019. Accessed January 10, 2024. https://www.un.org/en/development/desa/population/publications/pdf/family/familyPlanning_DataBooklet_2019.pdf

[8] Oraby D. Determinants of knowledge in relation to sexual and reproductive health of adolescents in the Middle East and North Africa Region. In: Barakat C, Dghaim R, Al Anouti F, eds. *Adolescent Health in the Middle East and North Africa: An Epidemiological Perspective.* Global Perspectives on Health Geography. Springer, Cham; 2022:95–104. 10.1007/978-3-030-92107-1_6

[9] Oraby DM. Sexual and reproductive health among young people in Egypt: the role and contribution of youth-friendly services. Sex Educ. 2013;13(4):470–477. 10.1080/14681811.2012.756810

[10] United Nations Population Fund (UNFPA). Arab States Regional Office (ASRO). *Between 3eib [*Shame in Arabic] and Marriage: Navigating Comprehensive Sexuality Education in the Arab Region: A Situational Analysis.* UNFPA ASRO; 2020. Accessed January 10, 2024. https://arabstates.unfpa.org/sites/default/files/pub-pdf/situational_analysis_final_for_web.pdf

[11] Nagi M. Islam, sexualities and education. In: Daun H, Arjmand R, eds. *Handbook of Islamic Education.* International Handbooks of Religion and Education, Vol 7. Springer, Cham; 2017:1–26. 10.1007/978-3-319-53620-0_11-1

[12] Tabatabaie A. Childhood and adolescent sexuality, Islam, and problematics of sex education: a call for re-examination. Sex Educ. 2015;15(3):276–288. 10.1080/14681811.2015.1005836

[13] Khan MA, Rassool GH, Mabud SA, Ahsan M. *Sexuality Education from an Islamic Perspective.* Cambridge Scholars Publishing; 2020.

[14] Ridanović Z. AIDS i islam [AIDS and Islam]. Article in Croatian. Med Arh. 1997;51(1–2):45–46. 9324573

[15] World Health Organization (WHO). Regional Office for the Eastern Mediterranean. *HIV Prevention and Care Among Injecting Drug Users in the Islamic Republic of Iran: A Review of Best Practice.* WHO Regional Office for the Eastern Mediterranean; 2006. Accessed January 10, 2024. https://applications.emro.who.int/aiecf/pre_care_among_injecting_en.pdf

[16] Kamarulzaman A, Saifuddeen SM. Islam and harm reduction. Int J Drug Policy. 2010;21(2):115–118. 10.1016/j.drugpo.2009.11.003. 20006483

